# MyDas, an Extensible Java DAS Server

**DOI:** 10.1371/journal.pone.0044180

**Published:** 2012-09-13

**Authors:** Gustavo A. Salazar, Leyla J. García, Philip Jones, Rafael C. Jimenez, Antony F. Quinn, Andrew M. Jenkinson, Nicola Mulder, Maria Martin, Sarah Hunter, Henning Hermjakob

**Affiliations:** 1 Computational Biology Group, Department of Clinical Laboratory Sciences, University of Cape Town, Cape Town, South Africa; 2 European Bioinformatics Institute, Hinxton, United Kingdom; University College Dublin, Ireland

## Abstract

A large number of diverse, complex, and distributed data resources are currently available in the Bioinformatics domain. The pace of discovery and the diversity of information means that centralised reference databases like UniProt and Ensembl cannot integrate all potentially relevant information sources. From a user perspective however, centralised access to all relevant information concerning a specific query is essential. The Distributed Annotation System (DAS) defines a communication protocol to exchange annotations on genomic and protein sequences; this standardisation enables clients to retrieve data from a myriad of sources, thus offering centralised access to end-users.

We introduce MyDas, a web server that facilitates the publishing of biological annotations according to the DAS specification. It deals with the common functionality requirements of making data available, while also providing an extension mechanism in order to implement the specifics of data store interaction. MyDas allows the user to define where the required information is located along with its structure, and is then responsible for the communication protocol details.

## Introduction

The integration of information is essential in any research project as a variety of data is generated in experiments, and must be analysed and validated in the context of the latest available knowledge-base. In Bioinformatics, this has proven to be a challenge, and for this reason multiple software solutions have been proposed, e.g. service oriented architectures, link integration, data warehousing, view integration, etc [1].

The Distributed Annotation System (DAS) is a widely used integration system in the bioinformatics field, and provides a method for taking data from more than one web-based resource, and displaying it in a single view. DAS allows data to be integrated from multiple, heterogeneous databases in a standardised and portable format [2]. In this way, laboratories and research groups adopting the protocol can easily share, visualise and compare their data. The DAS architecture is service-oriented and is comprised of three components: the Registry, Sources and Clients. The DAS Registry (http://www.dasregistry.org) acts as a directory providing information about registered sources. DAS Sources provide an interface to access and retrieve biological data, and DAS Clients, such as Dasty3 [3], Dalliance [4] and myKaryoView [5], provide a unified view of biological information, collecting data from multiple sources.

**Figure 1:**
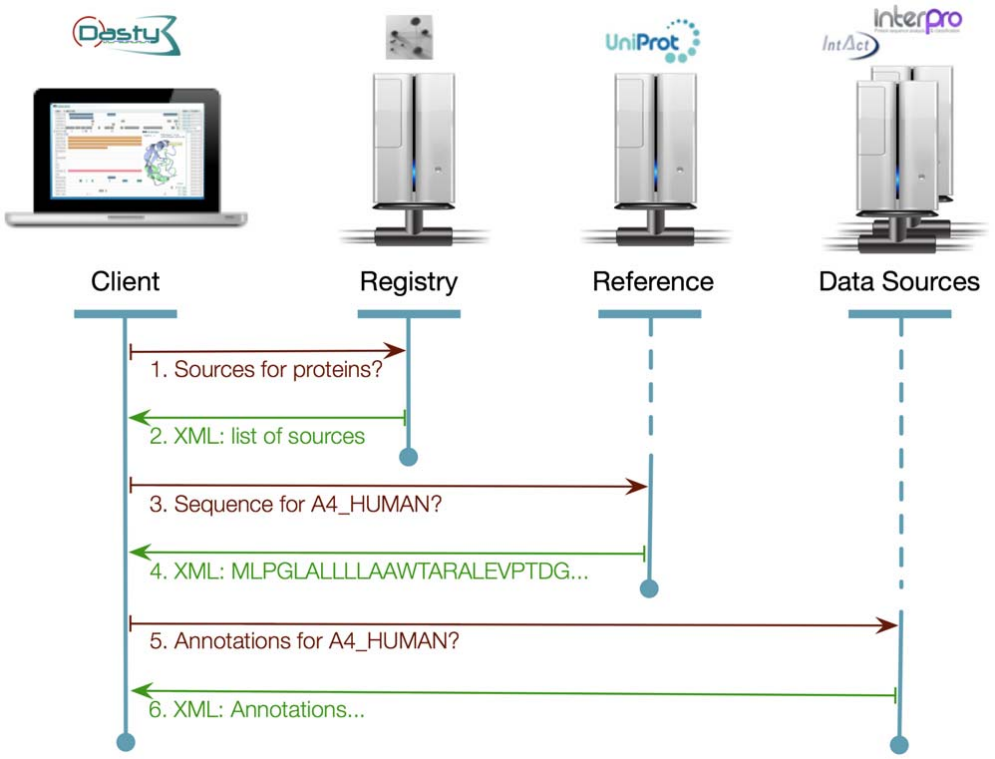
Flow of information in DAS. Interaction between a DAS client and the different DAS servers (i.e. Registry, Reference and Data Source).

A regular flow of information in DAS is shown in Figure 1. The DAS client requests information about a protein that can be specified by its accession number or identifier. The client then communicates with the DAS registry in order to retrieve a list of available sources providing information about that biological product. Once the client has retrieved this list, it proceeds to query the DAS reference source, i.e. a DAS source providing the sequence or structure of each molecule that it describes e.g. UniProt in the case of proteins. The DAS Reference source supplies not only the sequence but also meta-data such as the version. In this way clients can ascertain which retrieved annotations correspond to the original request. At this point, the client retrieves features, i.e. annotations, from the available DAS sources. These annotations may be applicable to specific subsections of the sequence (e.g. the location of active sites or observed peptides) or may be applicable to the entire sequence (e.g. related publications or taxonomy). Finally, the client organises and displays the annotations.

DAS servers may implement a number of different functions (called capabilities in DAS) depending on the information provided. The main DAS capabilities are:

- *Source:* A list of available data sources on the server.
- *Entry Points:* A list of molecule accession numbers described by a DAS source.
- *Sequence:* Corresponding to a given segment.
- *Types:* A list of annotation types available.
- *Features:* A list of annotations available for a segment.
- *Stylesheet:* Server recommendations on formatting the retrieved annotations.
- *Structure:* Protein structure, including metadata and coordinates.

At the beginning of 2011 the DAS Registry reached the milestone of 1000 data sources registered. Each of these data sources have to follow the DAS protocol, which involves the implementation of a wide variety of common functionality such as: parsing, capture of arguments, exception handling, XML creation, dealing with the HTTP protocol, and more. Each data source must also implement the interface with the actual data store. Ideally, implementing a new data source need not require the work of re-implementing this common functionality.

In this paper we introduce MyDas, a server that facilitates the publishing of biological data through the DAS protocol. This allows research groups to share their data in the context of existing information without requiring them to understand the full DAS Protocol. All they need to do is develop an adapter to specify how to access the raw data. This adapter is then used by MyDas to create the necessary HTTP interfaces to support all the DAS capabilities, such as querying by segment or recovering the types of annotations.

In the following sections we describe MyDas, how to use it, its architecture, existing alternatives, and present some examples of current DAS services built upon MyDas.

## Analysis

In a simplified way, MyDas is to DAS what the Apache Web Server is to the Web. When issued with a request, the output from Apache is typically an HTML file, whereas in MyDas the output is an XML document based on the DAS specification. In Apache, when a non standard response is required, it makes use of middleware technologies (such as PHP, JSP or ASP) to create a custom HTML document. In the case of MyDas, the functionality of the middleware can be defined through an adapter created by the owner of the information. This allows for superior customisation without the need for a detailed understanding of the protocol.

### Publishing Data With MyDas

When using MyDas to publish biological data, the following series of steps are suggested as a guide: define the type of source you want to publish; implement an adaptor to process this data; then configure and run MyDas in a web server. This allows the data to be accessed by a DAS client. Below is a more detailed explanation:

#### Define the type of the source

In the DAS protocol there are a number of types of source available, such as reference, annotation or alignment sources. MyDas allows the user to choose between several interfaces that represent those types, making sure the user is asked to implement the correct methods according to each type of DAS source.

For example a laboratory that works with well known proteins and is focused on discovering information about them does not need to publish sequences that are already available in the UniProt DAS source. Its requirement is to use the same identifiers in association with their existing coordinates and add further information, and therefore an annotation interface should be implemented. In contrast, a group working with de-novo sequencing techniques is interested in publishing the assembled sequences, and therefore publishing data as a reference source is more appropriate.

#### Create an adapter

A MyDas adapter is a Java class that represents the DAS source and provides an interface between the information source and the rest of the MyDas components. Some programming skills are required to create an adapter, as the nature and structure of the data varies from source to source. Depending on which interface has been selected in the previous step, different methods need to be implemented. MyDas then uses these to respond to DAS commands.

These methods allow MyDas access to the origin of the information (databases, text files, servers, etc.) and to map that information onto the DAS model. For example, if the information is stored in a database, the adapters indicate which tables correspond to the segments, and which field to its id, name, etc.

#### Configure and deploy

An instance of MyDas can run multiple data sources. There is a configuration file where the user indicates the location of the adapter and meta information about the source, such as the name, organism and version. General aspects of the server are also configurable in the same file (e.g. allow compression, include XSL stylesheets, etc.).

In order to publish the information, MyDas must be installed in an internet-visible machine, otherwise the data will only be locally accessible.

### Design and Implementation

MyDas is a Java Servlet Application accepting HTTP requests, typically with one request for each command in the DAS specification. Responses are valid XML documents. MyDas is a Java 1.6 application, and it runs on a Java servlet container such as Tomcat (http://tomcat.apache.org/) or Jetty (http://jetty.codehaus.org/jetty/).

**Figure 2:**
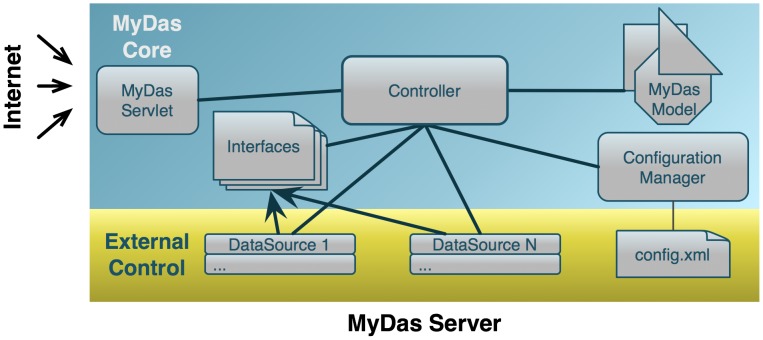
MyDas Architecture. The requesters can interact with MyDas through the servlet, which communicates the commands to the Controller. The Controller knows which Data Sources have been implemented by querying the Configuration Manager. Data Sources should implement at least one of the provided Interfaces. MyDas internally implements the DAS model.

Figure 2 illustrates the architecture of MyDas, separating the core (i.e. what is included in any installation) from external control (i.e. components that should be defined for each particular server as adapters to each particular kind of data). This division was created with the goal of providing DAS service developers with a platform of common methods to deploy their data as a DAS service. Every source has specific details such as its storage system (e.g. a relational database or flat file), and the strategy used to query the data (e.g. in-memory or using pre-indexing).

The configuration manager makes the user options available to both the MyDas core and the data source implementation. The configuration file is used to define the DAS sources, including data such as the URI, title, the relative path to the data source adapter and it also provides a method of defining general options such as the styles files (XSLT) to display the DAS data in a web browser.

MyDas includes a Java object model that represents the different elements that are retrieved by a DAS source. This must then be used by the data source developer when creating an adapter. In this way, MyDas is able to deploy heterogeneous data into DAS.

In order to facilitate the implementation of data sources, a template project is available, with examples of both reference and annotation servers.

## Results

### Showroom

MyDas is an extensible DAS server that has been designed with ease of development in mind. Where possible, MyDas uses the same terminology as in the DAS specification, with the consequence that DAS service providers will experience a shallow learning curve when implementing a MyDas DAS service for the first time. MyDas is being adopted by different data providers, including UniProt, InterPro and PRIDE.

UniProt (Universal Protein Resource) [6] is a comprehensive catalogue of protein sequences and functional information. It consists of different databases, each optimized for different uses. The UniProt Knowledgebase (UniProtKB) is an expertly curated database providing a central access point for integrated protein sequence information. The UniProt Archive (UniParc) is a non-redundant sequence repository of all publicly available protein sequences. UniProt DAS (http://www.ebi.ac.uk/das-srv/uniprot/das/uniprot, http://www.ebi.ac.uk/das-srv/uniprot/das/uniparc) acts as a reference and annotation server, providing access to up-to-date information and allowing queries by UniProtKB and UniParc accessions numbers. There are currently more than 50 Data Sources that use UniProt DAS as a reference.

The InterPro database of predictive protein signatures is used for the classification and automatic annotation of proteins and genomes [7]. InterPro provides several DAS data sources: DS_327 serves (http://www.ebi.ac.uk/das-srv/interpro/das/InterPro) matches that have been calculated to the predictive models supplied by the InterPro member databases for all UniProtKB protein sequences. DS_1028 (http://www.ebi.ac.uk/das-srv/interpro/das/InterPro-matches-overview) serves these matches resolved to the InterPro entries that integrate the member database signatures (providing a compact summary view of the domains, families and sites predicted for each UniProtKB sequence), and finally DS_1029 (http://www.ebi.ac.uk/das-srv/interpro/das/InterPro-UniParc-matches) serves matches to member database signatures that have been calculated for UniParc protein sequences.

PRIDE DAS 1.6 (http://www.ebi.ac.uk/pride-das/das/) provides protein and peptide identifications together with supporting mass spectrometry evidence [8]. The information from PRIDE has already been shared using BioMart [9], therefore the strategy used to make it public to the DAS community was to develop an adaptor using MyDas to take the information from this source.

### A Use Case Scenario

To aid in the understanding of the above steps, we have created a set of tutorials for MyDas that are accessible via web (http://code.google.com/p/mydas/wiki/Tutorials). The tutorials explain how to obtain different data examples and define the queries. Most importantly, they explain how to map the results of those queries onto the DAS model.

The power of MyDas is revealed when used on large data sets with elaborate schemas. The use of a database system is a common way to store/access data in bioinformatics environments. Take, for example, a laboratory that works with a Laboratory Information Management System (LIMS) to organize their information - all their data is already stored in a database. Many institutions may have a similar setup, however schema, policies and software vary from place to place. Although exporting files (and using them to publish data) is an option, it implies that changes in the database won't be reflected in the generated files. In contrast, MyDas can be set up to take the information directly from the database management system and therefore will always be up to date.

The main example used in the tutorials and demonstrated here, considers the freely available mysql database provided by Ensembl. There are over a hundred databases hosted on the Ensembl servers, and in this case we used the core set of tables for Homo sapiens (version 56_37a), and restricted our search scope to some high level features (e.g. Chromosome, genes, transcript).

**Figure 3:**
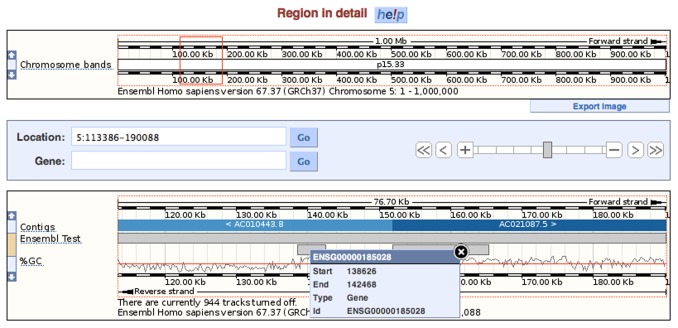
A MyDas Source as displayed on the Ensembl client. The data source created during the MyDas tutorials as it is visualised on the Ensembl web browser.

We used MyDas to publish this subset of the Ensembl database and make it queryable through DAS commands. A data source like this can be used by several tools to visualize its data. Figure 3 is a snapshot of the Ensembl browser, including the track named ‘Ensembl Test’, whose information is obtained from the tutorial data source, demonstrating how the data published with MyDas can be displayed in well known genome browsers.

### Other DAS Servers

There are other alternatives for publishing through DAS. For instance, easyDAS [10] is a preinstalled server, where a new data source can be configured by submitting a GFF file (or similar). Although this alternative is ideal for rapid publishing, it lacks granular control over the data once it is deployed. It is also limited to plain files, and therefore more advanced storage systems such as databases or indexed files are out of its scope.

Providers of biological information can use DAS servers like Dazzle (www.biojava.org/wiki/Dazzle), ProServer [11] or MyDas, amongst others, to set up their sources. However, MyDas and ProServer are the only servers that fully support the current DAS specification (1.6). They differ from each other mainly in the language in which they are implemented (ProServer is written in Perl), but not in feature set, making system compatibility the major factor in deciding between the two.

Table 1 summarizes some of the high level characteristics of the most well known DAS servers.

**Table 1:** Features of the main DAS servers.

*Feature*	*MyDas*	*ProServer*	*Dazzle*	*easyDAS*
Language	Java	Perl	Java	Web App(Perl)
Latest Release	2011	2011	2010	2011
DAS Version	1.6	1.6	1.53E*	1.6
Physical Storage	Defined by User	Defined by User	Defined by User	Internal database
Entity Responsible	EBI	Sanger Institute	Sanger Institute	EBI
Main task to create a data source	Develop a Java class.	Develop a Perl adaptor	Develop a Java class	Submit a tabulated file.

* There is a branch of this project where capabilities of DAS 1.6 are been implemented, however there was not a stable version of it at the time of publishing.

We prepared a stress test using the Apache HTTP Server Benchmarking Tool (http://httpd.apache.org/docs/2.0/programs/ab.html) to compare the loading performance of DAS servers. The servers were installed on the same machine, and the requests were triggered there too, with the purpose of avoiding any bias due to network conditions and computer specifications. easyDas was not taken into account for this test because it is installed on a different server, therefore there is no way to exclude network latency from the test.

Given that the three servers provide a Data Source implementation to publish data from a GFF file, they were configured to use the same GFF file to ensure equal conditions. While the implementation details of each adaptor may vary, the three examples were created with the goal of exemplifying the use of each server, giving more priority to understandability of the code than performance. Nonetheless, we consider this a fair test, because most users will use these examples as templates of how to implement a Data Source in each corresponding DAS server.

The test was designed to repeat the same query 1000 times with 10 concurrent connections. Three queries were executed, one to return a document of approximately 1500 bytes (small), a second one with 200000 bytes (medium), and one returning the whole file, which in DAS format is approximately 7200000 bytes (large).

The 3 servers were able to complete all the requests. However, the large test for ProServer was run on a different machine with similar specifications because of a local issue with the first computer. Table 2 shows the main results of the executed test.

**Table 2:** Benchmarking between the main DAS servers.

*Figure*	*MyDas*	*ProServer*	*Dazzle*
Requests per Second - Mean (small)	739.88	1.54	424.56
Time per request - Mean (small)	13.516 ms	6492.978 ms	23.554 ms
Transfer Rate (small)	1534.68 Kbytes/sec	2.81 Kbytes/sec	859.91 Kbytes/sec
Requests per Second - Mean (medium)	51.52	1.40	34.10
Time per request - Mean (medium)	194.114 ms	7123.396	293.216 ms
Transfer Rate (medium)	10944.96 Kbytes/sec	288.19 Kbytes/sec	6997.52 Kbytes/sec
Requests per Second - Mean (large)	1.79	0.32	1.10
Time per request - Mean (large)	5589.148 ms	30942.590 ms	9110.292 ms
Transfer Rate (large)	13088.38 Kbytes/sec	2283.04 Kbytes/sec	7770.29 Kbytes/sec

The figures in the table show that in all 3 scenarios MyDas performed better than the other servers. It is important to note that both MyDas and Dazzle were running on the same Tomcat server, therefore the conditions for both were the same. ProServer on the other hand, is a standalone server that implements socket communications in the application itself, which is an advantage in terms of making it easy to use.

The fact that the ProServer figures do not show much variation during the different tests leads us to believe that the adaptor implemented there analyses the whole file each time. This is not ideal for the purposes of a clear comparison, only the large test was really comparable with the other two servers since, in this case, all three instances must go through the whole dataset.

In that test, MyDas still performed better than ProServer and Dazzle. However the implications of this test are limited because the performances shown here are directly proportional to the level of optimization that each adaptor has, and none of the implementations have been particularly tuned with this purpose. The complete results of the tests are available in Appendix S1.

## Discussion

MyDas currently forms the basis for high volume DAS servers like UniProt and InterPro. It combines performance and stability with ease of installation, operation, and extension. The simplest way to run the server is to provide annotations in the form of a simple GFF file. At another level, the MyDas interface is efficient at implementing additional custom data sources, such as relational databases.

While the recently published easyDAS server provides a platform for DAS-based sharing of small sets of nucleic acid or protein annotations, and ProServer addresses Perl-based environments, MyDas offers a developer-friendly solution for laboratories and institutions that wish to share medium to large scale datasets in a Java-based environment. It completes the landscape of modern, open source DAS servers available to organisations sharing biomolecular data via the distributed DAS protocol.

### Availability

- Project name: MyDas
- Project home page: http://code.google.com/p/mydas/
- Source code repository: http://mydas.googlecode.com/svn/trunk/
- Operating system(s): Platform independent
- Programming language: Java
- Other requirements: Java 1.5 or higher, Tomcat 5.0 or other servlet server.
- License: Apache 2.0

## Supporting Information

Appendix S1 **Detailed Report of the Benchmarking Test.**
(PDF)
