# Supplementary material for: MyDas, an Extensible Java DAS Server
Source: PLoS One. 2012 Sep 13;7(9):e44180. doi: 10.1371/journal.pone.0044180 (PMC3441562; doi:10.1371/journal.pone.0044180)
Supplement: Appendix S1 — Detailed Report of the Benchmarking Test. (PDF) [file pone.0044180.s001.pdf]

Results of a stress test between DAS servers.

We prepared a stress test using the Apache HTTP Server Benchmarking Tool (http://httpd.apache.org/docs/2.0/programs/ab.html) to compare the loading performance of DAS servers.
Three servers were installed on the same machine: MyDas, ProServer and Dazzle.
The requests were triggered on the same machine, with the purpose of avoiding any bias due to network conditions and computer specs.

The test was designed to repeat the same query 1000 times with 10 concurrent connections. Three queries were used in the test, one to return a document of approximately 1500 bytes(small), a second one with 200000 bytes(medium), and one returning the whole file, which in DAS format is approximately 7200000 bytes(large).

Specifications of the machine used for the test

- Brand: Apple
- Model: Macbook pro a1211
- Processor: 2.16 GHz intel Core 2 Duo
- Memory: 3 GB 667MHz DDR2 SDRAM
- Operative System: Mac OS X 10.6.8 Snow Leopard

Data

The data set used for the tests corresponds with the annotations for the mitochondrial DNA of the organism *C. Elegans* freely available at the URL:
ftp://ftp.sanger.ac.uk/pub/wormbase/releases/WS220/genomes/c_elegans/genome_feature_tables/GFF2/CHROMOSOME_MtDNA.gff
The file contains 18510 annotations and is 3.4 MB in size. It is a text file whose format follows the GFF2 standard.

Software Installation

MyDas and Dazzle are Java servlet applications. The servlet container used for both was Apache Tomcat 7.0.11 and the Java Runtime Environment running on the machine is version 1.6.0_33.
ProServer requires perl and the version installed on the test machine is version v5.12.3.
The installation instructions of the software and its corresponding data sources can be found on the relevant server web sites:

- MyDas:
  http://code.google.com/p/mydas/wiki/MyDASTutorial
- ProServer:
  http://proserver.svn.sourceforge.net/viewvc/proserver/trunk/doc/proserver_guide.html#install
- Dazzle:
  http://biojava.org/wiki/Dazzle#Getting_Dazzle

Test execution and result files:

The results returned by the apache benchmarking tool during testing are shown below

- **Small test** (~1500bytes):
  - MyDas

```
Server Software:        Apache-Coyote/1.1
Server Hostname:        localhost
```

```
Server Port:            8080

Document Path:          /MyDasTemplate/das/examplegff/features?segment=CHROMOSOME_MtDNA:1,50
Document Length:        1554 bytes

Concurrency Level:      10
Time taken for tests:   1.352 seconds
Complete requests:      1000
Failed requests:        0
Write errors:           0
Total transferred:      2124000 bytes
HTML transferred:       1554000 bytes
Requests per second:    739.88 [#/sec] (mean)
Time per request:       13.516 [ms] (mean)
Time per request:       1.352 [ms] (mean, across all concurrent requests)
Transfer rate:          1534.68 [Kbytes/sec] received

Connection Times (ms)
              min  mean[+/-sd] median   max
Connect:        0    1   1.1      0      13
Processing:     2   13  11.2     10     112
Waiting:        0   12  11.1      9     112
Total:          2   13  11.3     10     112

Percentage of the requests served within a certain time (ms)
  50%     10
  66%     14
  75%     18
  80%     20
  90%     27
  95%     36
  98%     47
  99%     53
 100%    112 (longest request)
```

- ProServer

```
Server Software:        ProServer/756
Server Hostname:        localhost
Server Port:            9000

Document Path:          /das/mygff/features?segment=CHROMOSOME_MtDNA:1,50
Document Length:        1421 bytes

Concurrency Level:      10
Time taken for tests:   649.298 seconds
Complete requests:      1000
Failed requests:        0
Write errors:           0
Total transferred:      1865000 bytes
HTML transferred:       1421000 bytes
Requests per second:    1.54 [#/sec] (mean)
Time per request:       6492.978 [ms] (mean)
Time per request:       649.298 [ms] (mean, across all concurrent requests)
Transfer rate:          2.81 [Kbytes/sec] received

Connection Times (ms)
              min  mean[+/-sd] median   max
Connect:        0    0   5.9      0     186
Processing:  4256 6481 403.0   6453   11716
Waiting:     4255 6478 403.1   6447   11715
Total:       4256 6481 403.1   6453   11716

Percentage of the requests served within a certain time (ms)
  50%   6453
  66%   6621
  75%   6726
  80%   6782
  90%   6947
  95%   7064
  98%   7244
```

```
  99%   7353
 100%  11716 (longest request)
```

- Dazzle

```
Server Software:        Apache-Coyote/1.1
Server Hostname:        localhost
Server Port:            8080

Document Path:          /dazzle-webapp/tss/features?segment=CHROMOSOME_MtDNA:1,50
Document Length:        1605 bytes

Concurrency Level:      10
Time taken for tests:   2.355 seconds
Complete requests:      1000
Failed requests:        0
Write errors:           0
Total transferred:      2074000 bytes
HTML transferred:       1605000 bytes
Requests per second:    424.56 [#/sec] (mean)
Time per request:       23.554 [ms] (mean)
Time per request:       2.355 [ms] (mean, across all concurrent requests)
Transfer rate:          859.91 [Kbytes/sec] received

Connection Times (ms)
              min  mean[+/-sd] median   max
Connect:        0    1   1.7      0      22
Processing:     3   23  26.4     14     196
Waiting:        0   21  25.4     13     196
Total:          3   23  26.4     14     197

Percentage of the requests served within a certain time (ms)
  50%     14
  66%     21
  75%     27
  80%     32
  90%     52
  95%     80
  98%    115
  99%    135
 100%    197 (longest request)
```

- **Medium test** (~20000bytes):
  - MyDas

```
Server Software:        Apache-Coyote/1.1
Server Hostname:        localhost
Server Port:            8080

Document Path:          /MyDasTemplate/das/examplegff/features?segment=CHROMOSOME_MtDNA:1,200
Document Length:        216986 bytes

Concurrency Level:      10
Time taken for tests:   19.411 seconds
Complete requests:      1000
Failed requests:        0
Write errors:           0
Total transferred:      217556000 bytes
HTML transferred:       216986000 bytes
Requests per second:    51.52 [#/sec] (mean)
Time per request:       194.114 [ms] (mean)
Time per request:       19.411 [ms] (mean, across all concurrent requests)
Transfer rate:          10944.96 [Kbytes/sec] received

Connection Times (ms)
              min  mean[+/-sd] median   max
Connect:        0    0   1.1      0      19
Processing:    19  193 159.6    155    1305
Waiting:       11  180 161.5    138    1265
Total:         19  194 159.6    156    1305
```

```
Percentage of the requests served within a certain time (ms)
  50%    156
  66%    203
  75%    242
  80%    268
  90%    350
  95%    482
  98%    795
  99%    876
 100%   1305 (longest request)
```

- ProServer

```
Server Software:        ProServer/756
Server Hostname:        localhost
Server Port:            9000

Document Path:          /das/mygff/features?segment=CHROMOSOME_MtDNA:1,200
Document Length:        209768 bytes

Concurrency Level:      10
Time taken for tests:   712.340 seconds
Complete requests:      1000
Failed requests:        0
Write errors:           0
Total transferred:      210214000 bytes
HTML transferred:       209768000 bytes
Requests per second:    1.40 [#/sec] (mean)
Time per request:       7123.396 [ms] (mean)
Time per request:       712.340 [ms] (mean, across all concurrent requests)
Transfer rate:          288.19 [Kbytes/sec] received

Connection Times (ms)
              min  mean[+/-sd] median   max
Connect:        0    0   0.4      0      10
Processing:  5646 7116 220.2   7129    7836
Waiting:     5645 7112 220.5   7125    7835
Total:       5646 7116 220.2   7129    7836

Percentage of the requests served within a certain time (ms)
  50%   7129
  66%   7191
  75%   7228
  80%   7254
  90%   7336
  95%   7423
  98%   7612
  99%   7689
 100%   7836 (longest request)
```

- Dazzle

```
Server Software:        Apache-Coyote/1.1
Server Hostname:        localhost
Server Port:            8080

Document Path:          /dazzle-webapp/tss/features?segment=CHROMOSOME_MtDNA:1,200
Document Length:        209656 bytes

Concurrency Level:      10
Time taken for tests:   29.322 seconds
Complete requests:      1000
Failed requests:        0
Write errors:           0
Total transferred:      210103000 bytes
HTML transferred:       209656000 bytes
Requests per second:    34.10 [#/sec] (mean)
Time per request:       293.216 [ms] (mean)
Time per request:       29.322 [ms] (mean, across all concurrent requests)
Transfer rate:          6997.52 [Kbytes/sec] received
```

```
Connection Times (ms)
              min  mean[+/-sd] median   max
Connect:        0    0   1.1      0      11
Processing:    48  292 103.7    285    1023
Waiting:        6   35  47.9     15     441
Total:         48  293 103.6    285    1024

Percentage of the requests served within a certain time (ms)
  50%    285
  66%    319
  75%    348
  80%    368
  90%    409
  95%    461
  98%    541
  99%    612
 100%   1024 (longest request)
```

- **Large test** (~7000000bytes):
  - MyDas

```
Server Software:        Apache-Coyote/1.1
Server Hostname:        localhost
Server Port:            8080

Document Path:          /MyDasTemplate/das/examplegff/features?segment=CHROMOSOME_MtDNA:1,14000
Document Length:        7490285 bytes

Concurrency Level:      10
Time taken for tests:   558.915 seconds
Complete requests:      1000
Failed requests:        0
Write errors:           0
Total transferred:      7490855000 bytes
HTML transferred:       7490285000 bytes
Requests per second:    1.79 [#/sec] (mean)
Time per request:       5589.148 [ms] (mean)
Time per request:       558.915 [ms] (mean, across all concurrent requests)
Transfer rate:          13088.38 [Kbytes/sec] received

Connection Times (ms)
              min  mean[+/-sd] median   max
Connect:        0    0   1.4      0      15
Processing:  1786 5576 1905.6   5189   13877
Waiting:     1244 4856 1914.2   4446   13302
Total:       1786 5577 1905.7   5189   13877

Percentage of the requests served within a certain time (ms)
  50%   5189
  66%   6013
  75%   6452
  80%   6804
  90%   7900
  95%   9087
  98%  11267
  99%  12661
 100%  13877 (longest request)
```

  - ProServer

```
Server Software:        ProServer/756
Server Hostname:        localhost
Server Port:            9000

Document Path:          /das/mygff/features?segment=CHROMOSOME_MtDNA:1,14000
Document Length:        7233398 bytes

Concurrency Level:      2
Time taken for tests:   4541.578 seconds
Complete requests:      1000
```

```
Failed requests:        0
Write errors:           0
Total transferred:      7233845000 bytes
HTML transferred:       7233398000 bytes
Requests per second:    0.22 [#/sec] (mean)
Time per request:       9083.157 [ms] (mean)
Time per request:       4541.578 [ms] (mean, across all concurrent requests)
Transfer rate:          1555.47 [Kbytes/sec] received

Connection Times (ms)
              min  mean[+/-sd] median   max
Connect:        0    0   0.4      0       8
Processing:  7358 9079 602.1   9066   12428
Waiting:     7346 9065 601.1   9050   12403
Total:       7358 9079 602.1   9066   12428

Percentage of the requests served within a certain time (ms)
  50%   9066
  66%   9247
  75%   9421
  80%   9521
  90%   9774
  95%   9994
  98%  10452
  99%  10815
 100%  12428 (longest request)
```

- Dazzle

```
Server Software:        Apache-Coyote/1.1
Server Hostname:        localhost
Server Port:            8080

Document Path:          /dazzle-webapp/tss/features?segment=CHROMOSOME_MtDNA:1,14000
Document Length:        7248407 bytes

Concurrency Level:      10
Time taken for tests:   911.029 seconds
Complete requests:      1000
Failed requests:        0
Write errors:           0
Total transferred:      7248854000 bytes
HTML transferred:       7248407000 bytes
Requests per second:    1.10 [#/sec] (mean)
Time per request:       9110.292 [ms] (mean)
Time per request:       911.029 [ms] (mean, across all concurrent requests)
Transfer rate:          7770.29 [Kbytes/sec] received

Connection Times (ms)
              min  mean[+/-sd] median   max
Connect:        0    1   1.4      0      10
Processing:  5132 9094 444.3   9105   10426
Waiting:        0   16  21.2      8     200
Total:       5132 9094 444.3   9105   10427

Percentage of the requests served within a certain time (ms)
  50%   9105
  66%   9253
  75%   9358
  80%   9439
  90%   9599
  95%   9770
  98%   9935
  99%  10029
 100%  10427 (longest request)
```

Unfortunately because of a local issue, the execution of the large test with ProServer was limited to 2 concurrent queries, When more queries were used, the following error was shown:

```
apr_poll: The timeout specified has expired (70007)
```

We then decided to run the test on a different machine and it successfully ran the 10 concurrent connections giving the following results:

```
Server Software:        ProServer/756
Server Hostname:        localhost
Server Port:            9000

Document Path:          /das/mygff/features?segment=CHROMOSOME_MtDNA:1,10000000000
Document Length:        7233404 bytes

Concurrency Level:      10
Time taken for tests:   3094.259 seconds
Complete requests:      1000
Failed requests:        0
Write errors:           0
Total transferred:      7233851000 bytes
HTML transferred:       7233404000 bytes
Requests per second:    0.32 [#/sec] (mean)
Time per request:       30942.590 [ms] (mean)
Time per request:       3094.259 [ms] (mean, across all concurrent requests)
Transfer rate:          2283.04 [Kbytes/sec] received

Connection Times (ms)
              min  mean[+/-sd] median   max
Connect:        0    1   2.1      0      45
Processing:  5761 30889 5567.1  30507  116246
Waiting:     5745 30456 3662.3  30473   55656
Total:       5761 30890 5567.3  30511  116247
```

The machine used in this case has slightly better specifications, but the response time of ProServer was still longer than both Dazzle and MyDas. This leads us to conclude that even if the issues on the first machine are solved, the outcome of the comparison would have remained the same.
